# Etiology and Incidence of Viral and Bacterial Acute Respiratory Illness among Older Children and Adults in Rural Western Kenya, 2007–2010

**DOI:** 10.1371/journal.pone.0043656

**Published:** 2012-08-24

**Authors:** Daniel R. Feikin, M. Kariuki Njenga, Godfrey Bigogo, Barrack Aura, George Aol, Allan Audi, Geoffrey Jagero, Peter Ochieng Muluare, Stella Gikunju, Leonard Nderitu, Amanda Balish, Jonas Winchell, Eileen Schneider, Dean Erdman, M. Steven Oberste, Mark A. Katz, Robert F. Breiman

**Affiliations:** 1 Global Disease Detection Division, Centers for Disease Control and Prevention, International Emerging Infections Program, Kisumu, Kenya; 2 Kenya Medical Research Institute/Centers for Disease Control and Prevention Public Health and Research Collaboration, Kisumu, Kenya; 3 National Center for Immunizations and Respiratory Diseases, Centers for Disease Control and Prevention, Atlanta, Georgia, United States of America; Naval Medical Research Unit 6, United States of America

## Abstract

**Background:**

Few comprehensive data exist on disease incidence for specific etiologies of acute respiratory illness (ARI) in older children and adults in Africa.

**Methodology/Principal Findings:**

From March 1, 2007, to February 28, 2010, among a surveillance population of 21,420 persons >5 years old in rural western Kenya, we collected blood for culture and malaria smears, nasopharyngeal and oropharyngeal swabs for quantitative real-time PCR for ten viruses and three atypical bacteria, and urine for pneumococcal antigen testing on outpatients and inpatients meeting a ARI case definition (cough or difficulty breathing or chest pain and temperature >38.0°C or oxygen saturation <90% or hospitalization). We also collected swabs from asymptomatic controls, from which we calculated pathogen-attributable fractions, adjusting for age, season, and HIV-status, in logistic regression. We calculated incidence by pathogen, adjusting for health-seeking for ARI and pathogen-attributable fractions. Among 3,406 ARI patients >5 years old (adjusted annual incidence 12.0 per 100 person-years), influenza A virus was the most common virus (22% overall; 11% inpatients, 27% outpatients) and Streptococcus pneumoniae was the most common bacteria (16% overall; 23% inpatients, 14% outpatients), yielding annual incidences of 2.6 and 1.7 episodes per 100 person-years, respectively. Influenza A virus, influenza B virus, respiratory syncytial virus (RSV) and human metapneumovirus were more prevalent in swabs among cases (22%, 6%, 8% and 5%, respectively) than controls. Adenovirus, parainfluenza viruses, rhinovirus/enterovirus, parechovirus, and Mycoplasma pneumoniae were not more prevalent among cases than controls. Pneumococcus and non-typhi Salmonella were more prevalent among HIV-infected adults, but prevalence of viruses was similar among HIV-infected and HIV-negative individuals. ARI incidence was highest during peak malaria season.

**Conclusions/Signficance:**

Vaccination against influenza and pneumococcus (by potential herd immunity from childhood vaccination or of HIV-infected adults) might prevent much of the substantial ARI incidence among persons >5 years old in similar rural African settings.

## Introduction

Compared with other regions, the mortality rate among older children and adults remains several-fold higher in sub-Saharan Africa, where acute respiratory infections (ARI) are a leading cause of this high mortality, as well as associated morbidity [1]. However, data on etiologies and rates of ARI among persons ≥5 years old in Africa have been principally focused on few geographic areas and pathogens (e.g., pneumococcus, tuberculosis). Few studies have comprehensively examined the etiologies of ARI among Africans ≥5 years of age. More importantly, there is even less available data on disease incidence for specific etiologies, which can inform public health policies regarding treatment and prevention.

From population-based surveillance in rural western Kenya undertaken from 2007–2010, we report bacterial and viral etiologies of ARI by age group, hospitalization status, HIV-infection status and season. We also provide incidence by etiology, adjusted for health-care seeking and presence of pathogens in asymptomatic controls.

## Methods

### Ethical Review

Written informed consent was obtained for data and specimen collection at the clinics and households. For children <13 years of age, written informed consent was obtained from parents or guardians for specimen collection. For minors aged 13–17 years of age, written informed consent was obtained from parents or guardians and written assent from the minor him/herself for specimen collection. The protocol and consent forms were reviewed and approved by the Institutional Review Boards of KEMRI (#932) and CDC (#4566).

### Study Site

CDC’s International Emerging Infections Program and the Kenya Medical Research Institute (KEMRI) have conducted population-based, infectious disease surveillance since late 2005 in Asembo, Nyanza Province, in rural western Kenya [2], [3]. All households in 33 villages within 5 kilometers of the referral facility, St. Elizabeth Lwak Mission Hospital (Lwak Hospital), were offered enrollment –78% were enrolled. The surveillance population on July 1, 2007, included 21,420 persons ≥5 years old, who had resided permanently in the area for at least 4 calendar months [4], [5]. Enrollment was continuous since the project’s beginning. Malaria transmission was holoendemic [5], [6]. HIV prevalence was high (17% in adults ≥18 years in 2008) [7].

### Clinic Surveillance

From March 1, 2007, to February 28, 2010, all enrolled participants received free medical care by KEMRI/CDC-trained nurses and clinical officers at Lwak Hospital for acute illnesses [2], [3]. Lwak hospital has 40 inpatient beds and manages most hospitalizations, only referring complicated patients. No chest radiographs were taken during the study period.

ARI was defined, using a variation on the definition of severe acute respiratory illness suggested for influenza surveillance, as cough *or* difficulty breathing *or* chest pain *and* documented axillary temperature ≥38.0^o^C *or* oxygen saturation <90% *or* hospitalization [2], [8]. For ARI patients, blood, nasopharyngeal and oropharyngeal specimens using polyester-tipped swabs, and urine were collected. Giemsa-stained malaria blood smears were performed on patients with history of fever or documented temperature ≥38.0°C.

### Household Surveillance

Community interviewers visited enrolled households every two weeks and questioned participants using a standardized questionnaire, in the local language, about all illness episodes in the past two weeks, including symptoms and health-seeking [3]. For this analysis, we defined ARI from the household visits as cough, difficulty breathing or chest pain *and* reported fever. The ARI cases from the household visit were only used to assess health-seeking patterns in the area and not directly included in the incidence calculations. No specimens were collected at household visits.

### Control Selection

From January 1, 2009, asymptomatic controls were enrolled from Lwak Hospital. Eligible controls were those who presented with non-severe illness (i.e., not requiring hospitalization), for immunizations, or for medicine refills. Eligible controls could not have had fever, any respiratory symptoms or diarrhea in the past two weeks. Each month we attempted to enroll a targeted number of controls, frequency-matched to cases by age and HIV status. The monthly target was 23 controls aged ≥5 years old (six HIV-infected), yielding power to detect a significant difference in detection of a pathogen between 12% of cases and 5% of controls. Nasopharyngeal and oropharyngeal swabs were collected on controls.

### Laboratory Testing

Clinic nurses attempted to collect five to ten ml of blood for culture, which was inoculated into commercially-produced blood culture bottles and bacterial growth identified using standard methodology, which we have described previously (BACTEC™ Aerobic PLUS™, Becton Dickinson, Belgium) [9], [10].

Naso- and oropharyngeal swabs were placed together in 1 ml viral transport media without antibiotics and transported the same day at 2°C–8°C to KEMRI/CDC laboratories near Kisumu, approximately 60 km from Lwak Hospital, where each specimen was divided into four aliquots and stored at −70°C. In monthly batches, a frozen aliquot was transported on dry ice to the KEMRI/CDC laboratory in Nairobi, where lab technicians, blinded to case-control status, tested it after one freeze-thaw cycle, using quantitative real-time reverse transcription polymerase chain reaction (qRT-PCR). Nucleic acid was extracted from 100 µL aliquots of each sample using Qiagen’s QIAamp viral RNA minikit (Qiagen Inc, California), according to manufacturer's instructions. Prior to August 2008, TaqMan® Universal PCR Master Mix (Applied Biosystems, California) was used for qRT-PCR; later qRT-PCR was carried out using AgPath-ID™ One-Step RT-PCR Reagents (Applied Biosystems). Samples were tested for the presence of adenovirus, respiratory syncytial virus (RSV), human metapneumovirus (hMPV), influenza A and B viruses, and parainfluenza virus (PIV) types 1–3 using qRT-PCR assays using previously published assays [11], [12]. Each clinical specimen was also tested for the human ribonuclease P gene to measure nucleic acid integrity and to confirm sample adequacy. A qRT-PCR test result was considered positive if an exponential fluorescence curve was produced that crossed the assigned threshold at C_T_ <40.0 [12].

Between January 1, 2009, and February 28, 2010, enhanced testing was done, which included testing respiratory swabs for three additional viruses (rhinovirus, enterovirus, and parechovirus) and atypical bacteria, and testing of asymptomatic controls. For testing of swabs for these additional pathogens, total nucleic acid extracts were prepared from 100µl of specimen per aliquot using MagMAX Viral RNA Isolation Kit (Applied Biosystems) in Nairobi and transported to the Viral Respiratory Diseases laboratory at CDC Atlanta on dry ice. Singleplex qRT-PCR for rhinovirus, enterovirus and parechovirus were performed using previously published methodologies [13], [14], [15]. The rhino- and enterovirus assays targeted the 5′ noncoding region of an area of high sequence similarity between human rhinovirus and enterovirus and exhibit some cross-reactivity (S. Oberste, personal communication). Therefore, positive rhinovirus and/or enterovirus qRT-PCR were reported together as rhino/enterovirus positive. For atypical bacteria, multiplex qRT-PCR was performed at KEMRI/CDC laboratories in Nairobi for *Mycoplasma pneumoniae, Chlamydia pneumoniae* and pan-*Legionella* species using published assays [12], [16].

Urine specimens were tested for pneumococcal urine antigen using the BinaxNOW® kit (Binax Inc., Maine) starting in May 2007; kits were not consistently available throughout the entire study period. Sputum specimens were not collected by protocol, but only based on the clinician’s suspicion of pulmonary tuberculosis, and microscopy was done at Lwak Hospital using Ziehl-Neelsen staining.

HIV testing was performed as part of a home-based testing initiative during 2008 when all persons ≥13 years in the surveillance area were offered HIV testing (two parallel rapid HIV tests), as described previously [7]. Seventy-eight percent of eligible adults agreed to be tested. We assumed that a person’s HIV status during home-based testing was the same throughout the study period. HIV-testing was not performed routinely in the clinic on most patients during this period.

### Data Analysis

Structured questionnaires on scannable paper forms detailing the current illness were completed for all sick visits at Lwak Hospital. (TeleForm® software, Cardiff™, Vista, CA). At the household visits, data were collected using personal digital assistants (PDAs) [3].

Analysis was performed using SAS (version 9.2, Cary, NC). Proportions were compared using chi-square test or Fisher’s exact test, and medians by Wilcoxon Rank Sum test. Rate ratios and 95% confidence intervals were calculated using Fisher’s method (Computer Programs for Epidemiologists, PEPI, version 4.0x) for crude rates and the delta method for adjusted rates [17]. Correlation between monthly rates of ARI and percent positive for each pathogen were tested by Pearson’s or Spearman’s correlation coefficient, depending on normality of the data.

We compared detection of each virus by qRT-PCR between cases and asymptomatic controls. Odds ratios (OR) and 95% confidence intervals were calculated using unconditional logistic regression, adjusting for age group, season when the swab was taken (December-February, hot and dry; March-May, long rains; June-August, cooler and dry; September-November, short rains) and HIV status (positive, negative, unknown). We used the OR to calculate pathogen-attributable fractions, which estimated the proportion of cases positive for each virus in which the virus was the likely cause [18], [19]. The pathogen-attributable fractions were calculated as (OR-1)/OR; pathogen-attributable fractions were only calculated for viruses with ORs that were statistically significant (p<0.05). For purposes of this analysis, we assumed the pathogen-attributable fraction was 1.0 for *S. pneumoniae* based on the negligible probability of detection of *S. pneumoniae* in blood or urine of asymptomatic controls [20], [21].

**Figure 1:**
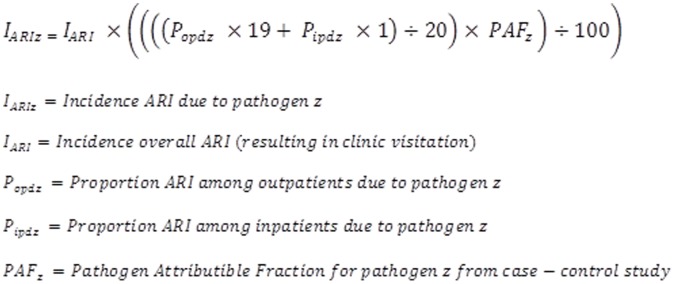
Equation to calculate incidence of ARI by etiology.

ARI incidence was calculated as the number of ARI clinic visits per 100 person-years of observation. Revisits for the same illness were not counted as separate episodes. Permanent residence status in the surveillance area was used to determine person-time contribution. Adjusted rates of clinic visitation were calculated accounting for the percentage of all clinic visits made for ARI that went to Lwak Hospital as opposed to other area clinics, as determined from the household visits [3]. Etiology-specific incidence was calculated by applying the proportions of each etiology to the adjusted incidence of ARI (Figure 1). Inpatients were over-represented among ARI patients tested at Lwak Hospital (35% inpatients), compared to ARI patients who visited a health facility in the community (5% inpatients), as determined by the household surveillance. Therefore, the inpatient and outpatient etiologic results from Lwak Hospital for each pathogen were weighted using a 19:1 outpatient:inpatient ratio. After adjusting for the outpatient/inpatient distribution, the pathogen-attributable fraction for each pathogen was applied to adjust the etiology-specific proportions. For those pathogens found to have an OR that included 1.0, no incidences were calculated because the role of the pathogen as a cause of ARI was not supported by our data.

## Results

During the study period, 3,406 ARI patients ≥5 years old were seen at Lwak Hospital, of whom 2,212 (65%) were outpatients and 1,194 (35%) were hospitalized. Among ARI patients, 2,978 (87%) had cough, 980 (29%) had difficulty breathing and 1,209 (35%) had chest pain; 2,602 (76%) had documented temperature ≥38.0°C, 188 (6%) had oxygen saturation <90% without fever (among a total of 242 with oxygen saturation <90%), and 616 (18%) were hospitalized without documented fever or low oxygen saturation. The majority (59%) was aged 5–17 years old and only 10% were ≥50 years old. Fifty-eight percent were female. Overall, 73 (2%) ARI patients died within 30 days of their clinic visit (0.2% among outpatients, 6% among inpatients). Among ARI patients ≥18 years old, 893 (65%) had HIV test results available, of whom 439 (49%) were HIV-infected, compared to 17% HIV-positivity among the entire surveillance population ≥18 years old [7].

### Bacterial Results

Among 3,406 ARI patients, 8.6% reported receiving one or more antibiotics before presentation (4.7% cotrimoxazole, 2.3% penicillin or amoxicillin, 2.0% other). Epidemiologic characteristics were similar between patients who did (n = 1,677, 49%) and did not (n = 1,729, 51%) have blood cultures taken (Table 1). The median blood volume for culture was 4.0 ml. Four percent of blood cultures grew contaminants (Table 2). Among uncontaminated cultures, the most common bacteria isolated were *Streptococcus pneumoniae* (3%) and non-Typhi *Salmonella* (3%). The percentage of patients with *S. pneumoniae* identified increased to 16% when adding urine antigen results to blood cultures (23% inpatients versus 14% outpatients, p = 0.003). Among the 131 patients who had *S. pneumoniae* detected, 10 (8%) were positive by blood culture only, 114 (87%) by urine antigen only, and 7 (5%) by both tests. The case-fatality ratio was higher among those with positive blood cultures for *S. pneumoniae* (7%) compared with those with only urine antigen detected (0.8%, p = 0.049). *S. pneumoniae* were detected more frequently among HIV-positive patients (28%) than HIV-negative patients (13%, p = 0.002).

**Table 1:** Comparison of demographics of persons who had blood cultures and nasopharyngeal (np) and oropharyngeal (op) specimens taken and those who did not among ARI patients, western Kenya, 2007–9.

	Blood culture			np/op specimens		
	Taken	Not taken	p value	Taken	Not taken	p value
N	1677	1729	0.37	1216	2190	
Age, median years (IQR)	13 (IQR = 24)	12 (IQR = 24)	0.11	12.0 (IQR = 23)	13.0 (IQR = 25)	0.75
Male, n (%)	721 (43)	707 (41)	0.23	522 (43)	906 (41)	0.40
Hospitalized, n (%)	595 (36)	599 (35)	0.63	396 (33)	798 (36)	0.03
HIV+, n (%)	257 (15)	232 (13)	0.03	179 (15)	310 (14)	0.03
HIV−, n (%)	365 (22)	437 (25)		255 (21)	547 (25)	
HIV unknown, n (%)	1055 (63)	1060 (61)		782 (64)	1333 (61)	
Died, n (%)	34 (2)	39 (2)	0.73	24 (2)	49 (2)	0.69

IQR is interquartile range.

**Table 2:** Etiologies of ARI by age group and pathogen, western Kenya. March 1, 2007,-February 28, 2010.

Age group	5–17 years	18–49 years	≥50 years	≥5 years	≥5 years CFR^a^ by pathogen	≥5 years, inpatient	≥5 years, outpatient	HIV positive ≥5 years	HIVnegative ≥5 years
Total patients seen	15979	10952	4271	31202	–	1939	29263	3,496	11,458
ARI cases	2024(13)	1041 (10)	341 (8)	3406 (11)	–	1194 (62)	2212 (8)	489 (14)	802 (7)
CFR^a^ for ARI cases	7(0.3)	51(5)	15 (4)	73(2)	–	69 (6)	4 (0.2)	20 (4)	6 (1)
**Bacteria**									
Blood cultures	965 (48)	556 (53)	156 (46)	1677 (49)	–	613 (51)	1082 (49)	257 (53)	365 (46)
without contaminant	924 (96)	544 (98)	149 (96)	1617 (96)	–	582 (95)	1035 (96)	249 (41)	353 (59)
*S. pneumoniae*	8 (0.9)^b^	23 (4)	10 (7)	41 (3)	3 (7)	27 (5)	14 (1)	15 (6)	8 (2)
Positive urine pneumococcal antigen^c^	68(13)	42 (17)	12 (20)	122 (15)	1 (0.8)	37 (19)	85 (13)	26 (23)	21 (12)
*S. pneumoniae* by blood culture or urine antigen^c^	68 (14)	47 (19)	16 (27)	131 (16)	2 (2)	45 (23)	86 (14)	30 (28)	22 (13)
*H. influenzae*	0	1 (0.2)	1 (0.7)	2(0.1)	0	2(0.3)	0	1 (0.4)	1 (0.3)
*S. aureus*	2 (0.2)	3 (0.6)	0	5 (0.3)	0	3 (0.5)	2 (0.2)	1 (0.4)	0
*Klebsiella pneumoniae*	0	2 (0.4)	1 (0.7)	3(0.2)	0	2 (0.3)	1 (0.1)	1 (0.4)	1 (0.3)
Nontyphi Salmonella	10(1)	27 (9)	4 (3)	41 (3)	0	16 (3)	25 (2)	14 (6)	6 (2)
Other pathogenic bacteria^d^	5(0.5)	8 (1)	2 (1)	15 (1)	1 (7)	9 (2)	6 (1)	2 (1)	5 (1)
**Viruses and atypical bacteria**									
Naso/orophangeal specimens	716 (35)	386 (37)	114 (33)	1216 (36)	–	396 (33)	820 (37)	179 (37)	255 (32)
Influenza A virus	169(24)	69 (18)	11 (10)	249 (20)	0	29 (7)	22(27)	27 (15)	53 (21)
Influenza B virus	45 (6)	21 (5)	4 (4)	70 (6)	0	8 (2)	62 (8)	9 (5)	20 (8)
RSV	57 (8)	28 (7)	7 (6)	92 (8)	3 (3)	33 (8)	59 (7)	17 (9)	13 (5)
Adenovirus	78 (11)	28 (7)	7 (6)	113 (9)	0	29 (7)	84 (10)	19 (11)	16 (6)
Parainfluenza virus 1	16 (2)	2 (0.5)	1 (1)	19(2)	0	2(0.5)	17 (2)	3 (2)	1 (0.4)
Parainfluenza virus 2	27 (4)	6 (2)	1 (1)	34 (3)	1 (3)	6 (2)	28(3)	4 (2)	5 (2)
Parainfluenza virus 3	46 (6)	17 (4)	6 (5)	69 (6)	1 (1)	14(4)	55 (7)	9 (5)	13 (5)
Human metapneumo	42 (6)	13 (3)	5 (4)	60 (5)	0	10 (3)	50 (6)	10 (6)	11 (4)
Rhino/Enterovirus^e^	109 (38)	29 (24)	9 (34)	147 (33)	3 (2)	39 (35)	108 (33)	13 (24)^e^	29 (32)^e^
Parechovirus^e^	2 (1)	0	0	2 (0.5)	0	0	2 (1)	0	0
Positive for ≥1 virus^e^	212 (73)	72 (59)	18 (53)	301 (68)	–	65 (58)	237 (71)	36 (67)	58 (64)
*M. pneumoniae* f	3 (1)	0	0	3 (1)	0	0	3 (0.5)	0	0

All data presented as number and percentage in parentheses rounded to nearest integer.

a Case-fatality ratios (CFR) are defined as death in the 30 days following clinic visit for ARI episode.

b Denominator for all calculation of percent of positive blood cultures is the number after removing contaminants – coagulase-negative *Staphylococcus*, *Bacillus* species, and corynebacterium.

c Only 841(25%) ARI patients total had urine collected for urine antigen testing for *S. pneumonia*, which started on May 21, 2007, and only 805 (24%) ARI patients had both blood culture and urine antigen testing for *S. pneumoniae* done. For HIV+and HIV−, 112 and 179 ARI patients had urine antigen testing, respectively, and 108 and 175 had both urine antigen and blood culture, respectively.

d Other pathogenic bacteria include *E. coli, Pseudomonas species, Moraxella catarrhalis,* group B *Streptococcus, Salmonella typhi*.

e Rhino/enterovirus and parechovirus were only tested for from January 1, 2009–February 28, 2010. 447 specimens were tested among persons ≥5 years old, of which 334 were from outpatients and 113 from inpatients, 290 among 5–17 year olds, 123 among 18–49 year olds and 34 among those ≥50 years old; 54 among HIV-positive individuals and 90 among HIV-negative persons.

f Atypical bacteria were detected by qPCR of np/op specimens. 561 specimens were tested for atypical bacteria. Besides *Mycoplasma pneumoniae*, the other atypical bacteria tested for were *Legionella pneumophila, Legionella* other species and *Chlamydia pneumoniae*. No positives were detected for any of these atypical bacteria.

*Mycoplasma pneumonia* was detected in 0.7% of patients; no *Legionella* or *Chlamydia pneumoniae* were detected (Table 2). Among 47 sputum samples tested by smear for *Mycobacterium tuberculosis*, 7 (15%) were read as positive.

### Viral Results

Epidemiologic characteristics were similar between patients who did (n = 1,216, 36%) and did not (n = 2,190, 64%) have naso/oropharyngeal swabs taken (Table 1). Overall during the time period of full viral testing, 68% of swabs were positive for at least 1 virus, detection being higher among outpatients (71%) than inpatients (58%, p = 0.008, Table 2). The detection of at least one virus decreased with increasing age (p<0.001). The most commonly detected virus was rhino/enterovirus (33%) followed by influenza A virus (22%). Detection of other viruses ranged from <1% to 9%. Influenza A virus was more often detected in outpatients (27%) than inpatients (11%, p<0.001) and among those <35 years old (23%) than those ≥35 years old (11%, p<0.001). Influenza B virus was also more common among outpatients (8%) than inpatients (2%, p<0.001). All other viruses were similarly detected among inpatients and outpatients. There were no significant differences in viral detection between HIV-positive and HIV-negative patients.

### Coinfection

Of 569 patients with full pathogen testing, 29% had >1 pathogen identified (Table 3). Detecting >1 pathogen was more common among HIV-positive patients (40%) than HIV-negative patients (25%, p = 0.04), but in similar proportions among inpatients (24%) and outpatients (31%, p = 0.21). The coinfection prevalence among those who had influenza (41%) was lower than that among those with other pathogens (66%, p<0.001). When limited to only those pathogens significantly more common in cases than controls, 18% of ARI patients had >1 pathogen identified; still no differences between inpatients (15%) and outpatients (19%) existed for coinfection (p = 0.51).

**Table 3:** Coinfection among ARI cases aged ≥5 years of age, limited to those that had full testing – nasopharyngeal/oropharyngeal specimens, blood culture and urine antigen testing for pneumococcus.

	Total	1 pathogen n (%)	2 pathogensn (%)	≥3 pathogensn (%)	Adenom/M^a^ (%)	RSVm/M^a^ (%)	hMPVm/M^a^ (%)	Influenzam/M^a^ (%)	Parainfl.m/M^a^ (%)	Rhino/enterom/M^a^ (%)	Parechom/M^a^ (%)	Pneumococcus m/M^a^ (%)
All	569	243 (43)	126 (22)	41(7)	48/64 (75)	28/46 (61)	31/37(84)	83/201 (41)	47/71(66)	55/104(53)	2/2(100)	52/76 (68)
HIV positive	58	20 (35)	19 (33)	4 (7)	5/11 (46)	3/6 (50)	6/6(100)	7/16(44)	6/9(67)	6/9(67)	0	11/12 (92)
HIV negative	121	51 (42)	24 (20)	6 (5)	3/7 (43)	1/5 (20)	5/5 (100)	16/49(33)	7/12(58)	13/23(57)	0	7/9 (78)

Coinfection defined as positive for another respiratory virus (from qPCR) or bacterial pathogen (from blood culture or pneumococcal urine antigen). January 1, 2009,–February 28, 2010, western Kenya.

a m = number co-infected with primary pathogen and at least one other viral or bacterial pathogen, M = total number infected with primary pathogen listed at top of column, % is the percent co-infected among persons with the primary pathogen.

### Case-control Results

Cases (n = 766) had a younger age distribution than controls (n = 273), with 68% and 42% aged 5–17 years old, respectively (p<0.01, Table 4). More cases (44%) than controls (33%) were enrolled in the hot, dry season (p<0.01). Among those with HIV-testing results available, 33% of cases and 39% of controls were HIV-positive (p = 0.28).

**Table 4:** Comparison of results of naso/oropharyngeal specimens between ARI cases and controls.

		Overall		Outpatient cases		Inpatient cases	
	Cases	Controls	OR (95% CI)	Cases	OR^a^ (95% CI)	Cases	OR^a^ (95% CI)
specimens, N	766	273	–	584	–	182	–
Influenza A virus	201 (26)	11 (4)	6.3 (3.3–11.9)	182 (31)	8.7 (4.5–16.8)	19 (10)	2.3 (1.1–5.2)
Influenza B virus	44 (6)	3 (1)	3.5 (1.1–11.8)	38 (7)	3.4 (0.98–11.4)	6 (3)	2.6 (0.62–11.1)
RSV	65 (8)	10 (4)	2.1 (1.0–4.3)	44 (8)	1.5 (0.71–3.4)	21 (12)	2.9 (1.3–6.6)
Adenovirus	86(11)	33 (12)	0.73 (0.46–1.2)	70 (12)	0.75 (0.45–1.2)	16 (9)	0.67 (0.35–1.3)
PIV1	16 (2)	0	0.98^b^	14 (2)	0.98^b^	2 (1)	0.99^b^
PIV2	31 (4)	3 (1)	3.3 (0.90–11.8)	26 (4)	2.4 (0.66–8.6)	5 (3)	3.6 (0.71–17.8)
PIV3	60 (8)	8 (3)	2.0 (0.91–4.3)	50 (9)	2.2 (0.98–5.0)	10 (5)	1.9 (0.69–5.0)
hMPV	49 (6)	5 (2)	2.6 (1.0–6.9)	42 (7)	3.0 (1.1–8.2)	7 (4)	1.6 (0.48–5.4)
*M. pneumoniae*	3 (0.5)^c^	1 (0.4)	0.51 (0.05–1.5)	3 (0.4)	0.57 (0.06–5.7)	0	0.99^b^
Rhino/Entero	147(33)^c^	44 (24)	0.95 (0.61–1.5)	108 (32)	0.76 (0.46–1.2)	39 (35)	1.5 (0.83–2.7)
Parechovirus	2 (0.5)^c^	1 (0.5)	0.90^b^	2	0.60^b^	0	–

January 1 2009, – Feb 28, 2010, western Kenya.

a For calculation of odds ratio (OR) for outpatient and inpatient cases, the same set of controls were used since controls were only outpatient.

b p value given when OR is not calculable.

c In this time period, specimens from 447 cases and 184 controls were tested were tested for *M. pneumoniae*, rhino/enterovirus and parechovirus. Among cases there were 334 outpatients and 113 inpatients.

After adjustment for age, season, and HIV status, detection of influenza A virus, influenza B virus, RSV and hMPV in the naso/oropharynx was associated with being a case (Table 4). Influenza A virus and hMPV were more strongly associated with outpatients than inpatients, although the confidence intervals overlapped. In contrast, RSV was associated with being an inpatient, but not an outpatient. PIV2 and PIV3 were also more common among cases, but did not reach statistical significance. Rhino/enteroviruses and adenovirus were equally common among cases (33% and 11%, respectively) and controls (24% and 12%, respectively).

### Seasonality

**Figure 2:**
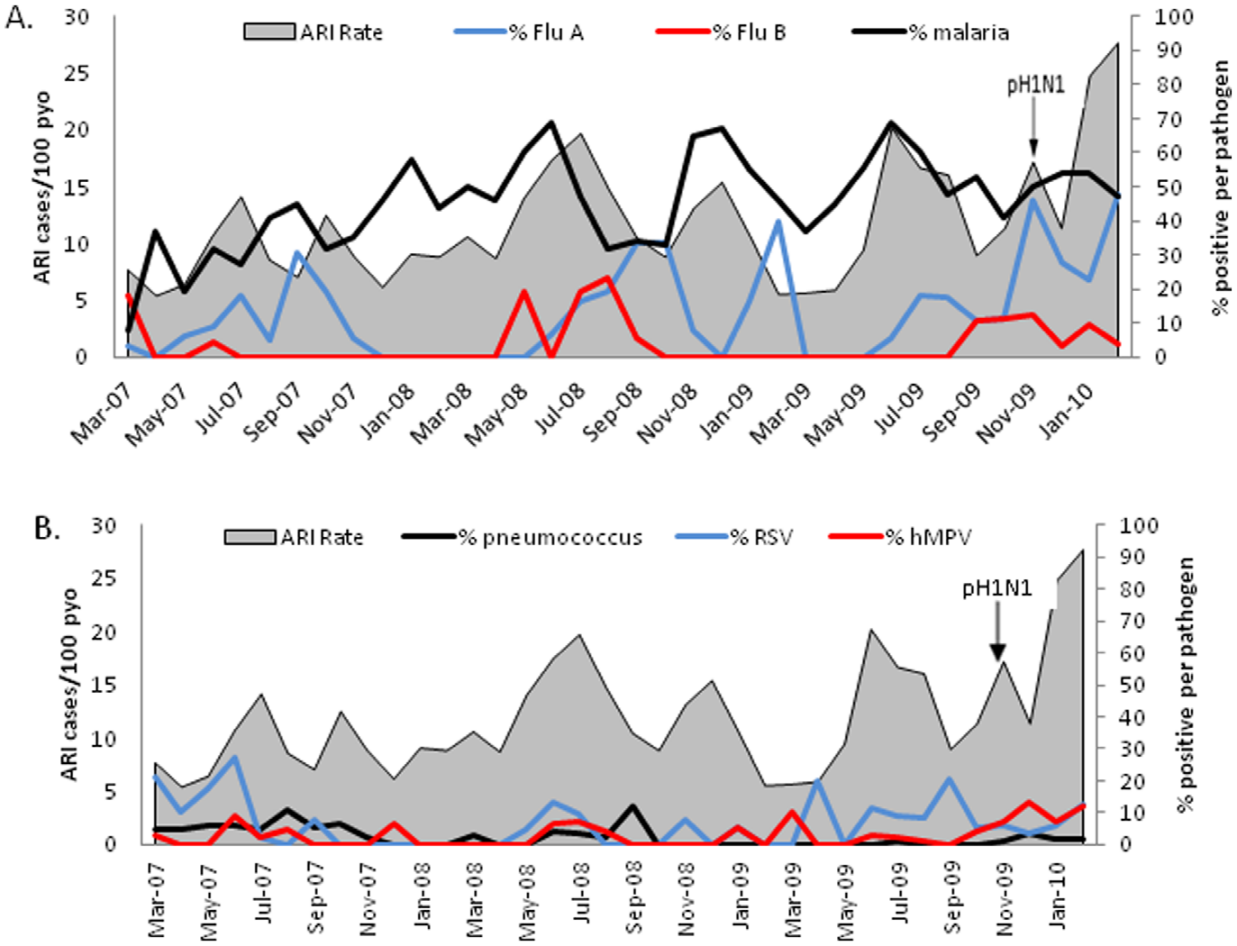
Monthly rates of ARI by etiology and malaria. The percent of ARI cases positive influenza viruses A and B is by qPCR and malaria by blood smear (panel A) and RSV and hMPV by qPCR and pneumococcus by blood culture (panel B), March 1, 2007-February 28, 2010. Asembo, western Kenya. Pneumococcus only given by blood culture because urine antigen testing was not available during entire time period. Pandemic H1N1 influenza virus (pH1N1) first detected at Lwak Hospital in November 2009.

The number of ARI cases tended to peak in June and July each year, with a smaller peak in October-December (Figure 2). There was a trend towards increasing ARI cases during the three year period (p = 0.002, linear regression). The number of ARI cases was highest in January-February 2010 when pandemic H1N1 influenza A virus circulated widely in Asembo [22]. Of the pathogens associated with case status in the case-control study, the monthly rate of ARI was correlated with the percentage positive for influenza A virus (p = 0.018), influenza B virus (p = 0.026), and hMPV (p = 0.012), but not for *S. pneumoniae* and RSV. Of note, when we limited the analysis to the 32 months prior to November 2009, when pandemic H1N1 virus first appeared, the monthly correlation between ARI case rates and viral respiratory pathogens was no longer significant. The percentage of malaria blood smears positive among ARI patients ranged from 8 to 69% by month, and tended to be higher in the rainy months (Figure 2). The monthly ARI rate was correlated with the malaria prevalence throughout the 36 months (p = 0.015), as well as in the 32 months prior to pandemic H1N1 (p = 0.007).

### Incidence

The adjusted overall incidence of ARI resulting in a clinic visit was 12.0 per 100 person-years for persons ≥5 years of age and 8.4 per 100 person-years for persons ≥18 years of age (Table 5). Among HIV-positive persons ≥18 years old, the rate of ARI was 21.4 per 100 person-years, compared to 5.6 for HIV-negative persons (RR 3.8, 95% CI 3.5–4.2). The highest pathogen-specific incidence among persons ≥5 years old was influenza A virus at 2.6 per 100 person-years, followed by pneumococcus at 1.7 per 100 person-years (Table 6). Rates of all pathogens were higher among HIV-positive than HIV-negative persons.

**Table 5:** Incidence rates of ARI for all persons ≥5 years of age, and among HIV-positive and HIV-negative persons ≥18 years from Lwak Hospital, western Kenya. March 1, 2007,-February 28, 2010.

	Overall Incidence			Incidence by HIV status^a^					
	pyo	Crude (95% CI)	Adjusted^b^(95% CI)	Crude			Adjusted^b^		
Age				HIV positive(95% CI)	HIV negative(95% CI)	RR (95% CI)	HIV positive(95% CI)	HIV negative(95% CI)	RR(95% CI)
5–17 years	19334	10.5 (10.0–10.9)	17.7 (16.9–18.5)	–	–	–	–	–	–
18–49 years	20666	5.0 (4.7–5.4)	8.8 (8.3–9.3)	11.9 (10.7–13.2)	2.9 (2.5–3.3)	4.1 (3.6–4.9)	20.3 (18.2–22.4)	4.9 (4.3–5.5)	4.2 (3.7–4.6)
≥50 years	9836	3.5 (3.1–3.9)	7.1 (6.3–7.8)	10.9 (8.4–13.8)	3.4 (2.9–3.9)	3.3 (2.5– 4.3)	21.0 (15.9–26.1)	6.4 (5.5–7.3)	3.2 (2.7–4.0)
≥5 years	49836	6.8 (6.6–7.1)	12.0 (11.6–12.4)	–	–	–	–	–	–
≥18 years	30502	4.5 (4.3–4.8)	8.4 (7.9–8.8)	11.8 (10.8–12.9)	3.1 (2.8–3.4)	3.8 (3.4–4.4)	21.4 (19.4–23.4)	5.6 (5.1–6.1)	3.8 (3.5–4.2)

Incidence is given as episodes per 100 person-years of observation (pyo).

a HIV status was known for 69% of the surveillance population ≥18 years old. Only those with known HIV status were included in the numerators and denominators for rate calculations by HIV status. For persons 5–12 years of age, HIV testing was not routinely done as part of home-based counseling and testing and therefore rates for 5–17 year olds is not included in rate calculations (see methods).

b Adjusted clinic rates were calculated by extrapolating from persons with ARI defined at household visit who sought care at another clinic besides the designated referral clinic, Lwak Hospital (see methods). In the age categories 5–17 years, 18–34 years, 35–49 years, ≥50 years, ≥5 years and ≥18 years, the percentage of ARI patients who sought care at another clinic besides Lwak were 41%, 43%, 43%, 48%, 53%, 43%, and 46% respectively. Health-seeking at Lwak was similar among HIV-positive and HIV-negative persons, so the same adjustment percentages were used.

**Table 6:** Incidence of select pathogens found to have an association with ARI case status, western Kenya. March 1, 2007,-February 28, 2010.

		All persons ≥5 years old		HIV positive ≥18 years old		HIV negative ≥18 years old	
	PAF^a^	Adjusted % ARI^b^	Rate per 100 pyo (95% CI)	Adjusted % ARI^b^	Rate per 100 pyo (95% CI)	Adjusted % ARI^b^	Rate per 100 pyo (95% CI)
Influenza A virus	0.84	21.7	2.60 (2.28–2.92)	21.0	4.49 (2.80–6.18)	25.4	1.42 (1.04–1.80)
Influenza B virus	0.71	1.6	0.20 (0.15–0.25)	4.3	0.92 (0.32–1.52)	7.8	0.44 (0.25–0.63)
RSV	0.52	3.7	0.44 (0.35–0.53)	4.6	0.98 (0.51–1.45)	2.3	0.13 (0.59–2.01)
hMPV	0.62	3.6	0.43 (0.32–0.54)	5.1	1.09 (0.41–1.77)	1.8	0.10 (0.04–0.16)
*S. pneumoniae*	1.0	14.5^c^	1.73 (1.43–2.03)	31.3	6.71 (4.31–9.11)	9.4	0.52 (0.30–0.74)

a The pathogen-attributable fraction (PAF) is calculated as (OR-1)/OR using viral prevalence data from cases and controls. For pneumococcus, AFE was assumed to be 1.

b The percentage of ARI was adjusted for the PAF and then adjusted for the prevalence among inpatients and outpatients for each pathogen at Lwak hospital and the percentage of ARI cases in the community who were seen in clinics as inpatients and outpatients (see methods).

c Only persons with complete testing for *S. pneumoniae* including blood culture and urine antigen testing were used to calculate the percent of ARI due to *S. pneumoniae*.

## Discussion

Our study was unique in several respects. First, we compared etiologies in inpatient and outpatient settings, whereas most previous adult etiology studies in Africa have focused on severely ill, hospitalized patients [23], [24], [25], [26]. We demonstrated different etiologic predominance for the same broadly defined ARI syndrome based on hospitalization status, with pneumococcus more common among inpatients and influenza viruses and hMPV among outpatients. Second, we enrolled a group of asymptomatic controls. As has been noted elsewhere, a positive result from PCR is not necessarily conclusive as to etiology because of its high analytic sensitivity; PCR may detect small amounts of nucleic acid due to past or asymptomatic infections [27], [28], [29], [30]. By enrolling asymptomatic controls, we were able to calculate a pathogen-attributable risk, which suggested that several viruses commonly found among ARI cases (i.e., rhino/enterovirus and adenovirus), were not associated with illness. Third, because we embedded our etiologic study in ongoing population-based surveillance, we were able to calculate incidence of etiology-specific ARI. The accuracy of our rate calculation was likely improved by adjusting for several factors that are often neglected, namely health-seeking patterns in the population and the attributable fraction of illness for each pathogen. Using the calculated incidence, we estimated that approximately 3% and 2% of adults in Nyanza Province (approximately 4.5 million persons ≥5 years, 2009), where Lwak is located, will have an episode of ARI from influenza viruses and pneumococcus each year, respectively, which translates to approximately 135,000 and 90,000 illnesses, respectively, in the province [31].

As expected, pneumococcus was the most common bacterial cause of serious ARI in this population, and was particularly prevalent among HIV-infected persons [2], [25], [32], [33], [34], [35], [36]. There was more than a 5-fold increase in the frequency of positive results for pneumococcus when incorporating the urine antigen test, supporting previous evidence that most pneumococcal pneumonia is non-bacteremic [25], [37]. Non-typhi *Salmonella* (NTS) was the second most common bacterium identified in ARI patients. NTS bacteremia commonly presents with respiratory symptoms in children, although its role in causing pneumonia based on lung aspiration studies is doubtful [25], [38]. Alternatively, patients could have a mixed infection where NTS bacteremia is accompanied by another pathogen causing ARI, particularly among HIV-infected persons who are at increased risk for both [23], [25], [38], [39].

The role of atypical bacteria as a cause of ARI in African adults is unclear. In South Africa, *C. pneumoniae* and *Legionella pneumophila* were detected by seroconversion in 21% and 9% of adults with pneumonia [40]. In Botswana, 17% of mostly HIV-infected adults had evidence for *M. pneumoniae* infection [24]. Apart from these studies, other studies of adults in less developed African countries showed low prevalence of atypical bacteria, a finding supported by our study [25], [34], [41], [42].

Influenza A virus was the most commonly detected virus. A prominent role of influenza viruses as a cause of ARI in Africa is becoming clear as more surveillance data becomes available [22], [43]. We found influenza viruses more often in outpatients than inpatients. We have previously speculated that even when detected among inpatients with ARI, influenza viruses might not be the primary reason for the admission [44].

Several other viruses besides influenza virus were associated with ARI. We found RSV associated with ARI among inpatients, but not outpatients. RSV has been shown to be the leading cause of hospitalized ARI in African children [45]. RSV has a less clear etiologic role among African adults. One study among South African adults did not find an excess in hospitalizations or mortality during RSV seasons [46]. In the U.S., several studies have postulated that RSV can cause pneumonia in adults, especially those with underlying disease or the institutionalized elderly, although none of these studies evaluated asymptomatic controls [47], [48]. In contrast to RSV, we found hMPV associated with ARI among outpatients, but not inpatients. The only study of hMPV among adults on the African continent was in Egypt and found hMPV in 14% of hospitalized adults with lower respiratory tract infections, but no asymptomatic controls were included [49].

Coinfection was common, with almost one-third of ARI patients having >1 pathogen identified. Coinfection could represent a true biologic phenomenon where infectious cofactors are necessary for pathogenesis. Alternatively, the frequency of coinfection could reflect the fact that certain pathogens, such as rhino/enteroviruses and adenoviruses, are commonly detected in the upper airways, with unclear clinical significance. Of note, ARI patients with influenza viruses detected had less coinfection, which might be related to the high pathogen attributable risk of influenza viruses and the lack of need for a coinfection to cause illness.

Our study cannot be considered a comprehensive assessment of the etiologies of ARI. Additional specimens likely would have increased the yield of pathogens identified. Lung aspirates have been shown to have high diagnostic yield in African children, and one study in African adults, as they sample material directly from the affected sections of the lung [25], [50], [51]. However, lung aspirates are rarely performed in African adults with pneumonia due to the potential risk of pneumothorax in populations with high risk of *Pneumocystis jiroveci* pneumonia (PCP). Bronchoscopy with bronchoalveolar lavage has been used in a few hospitals in Africa, and has improved diagnostic yield, although its use is usually reserved for severely ill, hypoxic patients in tertiary hospital settings [23], [25], [26], [34], [36]. While good-quality sputum specimens might have a role in diagnosis of some etiologies, if strict criteria for assigning a predominant organism are used, sputum is generally considered too prone to contamination with upper respiratory tract secretions to be useful diagnostically [30], [37], [52], [53]. Lack of these additional specimens limited our detection of *Pneumocystis jiroveci* and *Mycobacteria tuberculosis*, two important causes of severe respiratory infection in high HIV prevalence populations in Africa [23], [25], [26], [34], [36], [54], [55], [56]. Moreover, we did not test for some viruses, such as coronaviruses and bocavirus, which might play a role in pneumonia, although their pathogenicity, particularly in adults, is still debated [11], [34], [57], [58].

While we did not observe discrete seasonality of ARI in western Kenya as previously shown in temperate climates, there tended to be yearly peaks coincident with the peak malaria season, which usually occur 1–2 months after the rainy season begins. There could be several explanations for this. Malaria infection could augment the risk for ARI. An association between malaria and bacteremia in African children has been shown, although no direct associations between malaria and pneumonia in children or adults has been proven [59]. Second, similar climactic conditions could favor both malaria and pneumonia. We have shown previously that influenza incidence tends to be highest in Kenya during a broad wave that mostly corresponds with the southern hemisphere winter, from June to October, a time period that also encompasses peak malaria season [5], [22]. Lastly, malaria might cause symptoms that meet the nonspecific ARI case definition we used. The overlap in symptoms between malaria and pneumonia in children is well-documented [60]. Although similar evidence does not exist for older persons, the non-specificity of the ARI definition we used suggests that symptomatic malaria could have resulted in an illness that met the ARI case definition.

Our study had several other limitations. First, not all ARI patients were sampled. Reasons for not collecting swabs included high patient volume, after hours clinic attendance, and rare refusals (<10%). While we showed similar demographic characteristics and case-fatality ratios between sampled and non-sampled patients, undetected sampling bias might still have occurred that could influence the pathogens detected. Second, we used a broad ARI definition that likely captured a range of illnesses from influenza-like illness to pneumonia. Without further diagnostic procedures, such as chest radiographs, we could not confirm the anatomic location of the respiratory infection. Third, although the controls were by definition asymptomatic in the two weeks prior to enrollment, they might have been in the incubation period of a viral infection and subsequently developed symptoms. If this were true, a small number of controls might have been inaccurately classified. Fourth, unlike in developed countries, we found lower rates of ARI among the elderly, which we speculate is due to lower healthcare utilization by the elderly in rural Kenya [3], [61], [62]. Fifth, we assumed that a person’s HIV status during home-based testing in 2008 was unchanged throughout the study period, which might have led to some misclassification of HIV status. However, we feel that the number of misclassified individuals is small because the annual incidence of HIV among adults in the area has been estimated at 1% (KEMRI/CDC unpublished data), which at the most would have resulted in 39 individuals among the 1,291 ARI patients with HIV results having misclassified HIV status for some period of the three year surveillance period. Lastly, despite the lack of a statistical association with ARI at the population level for some viruses, an etiologic role of a virus detected in any given individual case cannot be ruled out. A study in Thailand showed that rhinovirus was associated with hospitalized respiratory illness among adults, particularly among the elderly [63]. Outbreaks of rhinovirus-associated pneumonia have been suspected among the elderly in developed countries [64]. Adenovirus has on occasion been implicated as a cause of pneumonia in African adults, but without a control group for comparison [65].

Our study demonstrated several opportunities to make an impact on the incidence of ARI among older children and adults in Africa. The high incidence among HIV-infected persons suggests that expanded testing and access to cotrimoxazole prophylaxis and anti-retroviral drugs will likely decrease the number of ARI cases, as has been shown before for pneumococcal infections [66], [67]. Vaccines could also decrease the incidence. Pneumococcal conjugate vaccine was introduced for infants in Kenya in early 2010 and if herd immunity occurs, as it has in developed countries, then decreases in adult pneumococcal disease incidence are expected [68]. Moreover, targeted vaccination among high-risk groups, such as HIV-infected adults, might be applicable for both influenza and pneumococcus [22], [32].
